# Mutation in ribosomal protein S5 leads to spectinomycin resistance in *Neisseria gonorrhoeae*

**DOI:** 10.3389/fmicb.2013.00186

**Published:** 2013-07-10

**Authors:** Elena N. Ilina, Maya V. Malakhova, Ivan N. Bodoev, Nina Y. Oparina, Alla V. Filimonova, Vadim M. Govorun

**Affiliations:** Research Institute of Physico-Chemical MedicineMoscow, Russia

**Keywords:** spectinomycin resistance, spot-transformation, horizontal gene transfer, ribosomal proteins, mutations

## Abstract

Spectinomycin remains a useful reserve option for therapy of gonorrhea. The emergence of multidrug-resistant *Neisseria gonorrhoeae* strains with decreased susceptibility to cefixime and to ceftriaxone makes it the only medicine still effective for treatment of gonorrhea infection in analogous cases. However, adoption of spectinomycin as a routinely used drug of choice was soon followed by reports of spectinomycin resistance. The main molecular mechanism of spectinomycin resistance in *N. gonorrhoeae* was C1192T substitution in 16S rRNA genes. Here we reported a Thr-24→Pro mutation in ribosomal protein S5 (RPS5) found in spectinomycin resistant clinical *N. gonorrhoeae* strain, which carried no changes in 16S rRNA. In a series of experiments, the transfer of *rpsE* gene allele encoding the mutant RPS5 to the recipient *N. gonorrhoeae* strains was analyzed. The relatively high rate of transformation [ca. 10^−5^ colony-forming units (CFUs)] indicates the possibility of spread of spectinonycin resistance within gonococcal population due to the horizontal gene transfer (HGT).

## Introduction

*Neisseria gonorrhoeae* is an obligate pathogen causing gonorrhea, one of the most abundant sexually transmitted diseases. The expansion of drug resistant *N. gonorrhoeae* strains is one of the global contemporary problems. The emergence and spreading of multidrug resistant (MDR) strains, which are resistant to penicillin, tetracycline, and ciprofloxacin, are reported throughout the world. In some countries, including Russia, about 50% of clinical *N. gonorrhoeae* strains identified as MDR (Kubanova et al., 2010; Allen et al., 2011). Until recently, all these strains remain susceptible to spectinomycin and to extended-spectrum cephalosporins (ceftriaxone and cefixime). Recently, *N. gonorrhoeae* strains displayed reduced susceptibility to the extended-spectrum cephalosporins. Moreover, several cases of clinical failures during the cefixime treatment have been reported (Wang et al., 2003; Heymans et al., 2012; Unemo et al., 2012). The spectinomycin remains the only antibiotic still effective in analogous cases. However, adoption of spectinomycin as the routinely used drug of choice was soon followed by reports of spectinomycin resistance (Boslego et al., 1987). The treatment of gonorrhea infection caused by extremely drug resistant (XDR) *N. gonorrhoeae* strains is very difficult, and the extended knowledge of molecular mechanisms of drug resistance is required for development of new tests needed to for routine diagnose in clinical practice.

In general, these are several mechanisms occurring in the bacteria which confer them antibiotic resistance. The most common are known to be drug inactivation, efflux-pumping of the drug out of the cell, and target modification due to single nucleotide change polymorphisms (SNPs) mainly (Davies and Davies, 2010). A bacterial strain can acquire resistance either by mutation of its own genes or by the uptake of exogenous genes by horizontal transfer from other microbes. Within bacterial population horizontal gene transfer (HGT) occurs via conjugation, transformation and transduction. With regards to neisseria genus these bacteria are naturally transformable and are capable to exchange their genetic material with high frequency (Koomey, 1998). This property leads to the rapid dissemination of antibiotic resistance markers and to the panmictic structure of the gonococcal and meningococcal populations.

Spectinomycin belongs to an aminocyclitol antibiotic class which blocks biosynthesis of bacterial proteins. After entering the bacterial cells, spectinomycin binds a ribosome beneath the 34 helix of 16S rRNA and interrupts elongation of the polypeptide during protein synthesis apparently preventing the translocation of the peptidyl tRNA from the A-site to the P-site (Carter et al., 2000; Borovinskaya et al., 2007).

Various bacteria demonstrate spectinomycin resistance, which results from three different mechanisms. The most frequent mechanism is the drug inactivation by adenylylation. Until now a diverse number of adenyltransferases, which exhibit the spectinomycin resistant (Spt-R) phenotype, was described (Shaw et al., 1993). One group of enzymes referred as AAD(3″)(9) [or ANT(3″)(9)] confers combined resistance to spectinomycin and streptomycin. These enzymes were found in a variety of gram-negative bacteria (Yamada et al., 1968; Hollingshead and Vapnek, 1985; Kehrenberg et al., 2005) and also in gram-positive bacteria (Clark et al., 1999). The other group of adenyltransferase referred as AAD(9) [or ANT(9)] confers the Spt-R phenotype only (LeBlanc et al., 1991).

On the other hand, the spectinomycin resistance can result from alteration of 30S subunit of bacterial ribosome due to mutations in chromosomal genes encoding ribosomal RNAs or proteins. Thus, mutations in the spectinomycin binding region of helix 34 of 16S rRNA encompassing the cross-linked positions from 1063 to 1066 and from 1190 to 1193 (in *Escherichia coli* numbering) lead to high level resistance to spectinomycin (Sigmund et al., 1984; Brink et al., 1994). These mutations were discovered for various bacteria such as *Chlamydia psittaci, Borrelia burgdorferi, E. coli, Pasteurella multocida* (Johanson and Hughes, 1995; Binet and Maurelli, 2005; Criswell et al., 2006; Kehrenberg and Schwarz, 2007) including *N. gonorrhoeae* and *N. meningitidis* (Maness et al., 1974; Galimand et al., 2000). Although ribosomal protein S5 (RPS5) is not involved in spectinomycin binding, it is located very close to the antibiotic binding site (within 5 A°) (Wirmer and Westhof, 2006). Accordingly, it has been found that mutations in RPS5 can lead to spectinomycin resistance in *E. coli* (Funatsu et al., 1972; Bilgin et al., 1990) and in *P. multocida* (Kehrenberg and Schwarz, 2007).

Until recently, the only 16S rRNA substitutions were found in Spt-R bacteria from *Neisseria* genus (Maness et al., 1974; Galimand et al., 2000). In this study, we reported the discovery of isolated mutation in RPS5 in Spt-R clinical *N. gonorrhoeae* strain, confirmed by Unemo et al. (2013), and estimate the potential of Spt-R horizontal spread. We established that the 10^−5^ colony-forming units (CFU) from transformation experiments represents a high risk of spectinomycin resistance spread within gonococcal population.

## Materials and methods

### Bacterial strains

*N. gonorrhoeae* clinical isolates were collected as a part of previous study (Ilina et al., 2008) and stored at a temperature of −80°C. The studied *N. gonorrhoeae* clinical isolates and strains are shown in Table 1.

**Table 1:** **Characteristics of gonococci involved into current investigation**.

**Name**	**Description**	**MICs (mg/L) for**					**Serotype**	**NG-MAST sequence type**
		**PEN**	**TET**	**CIP**	**SPT**	**CRO**		
e03.04	Clinical isolate	0.015	<0.03	0.001	32–64	<0.002	P1B3	5187
NG3	Clinical strain derived from e03.04 isolate	0.015–0.03	0.03–0.06	0.001–0.002	4–8	0.0005–0.001	P1B3	5187
NG3.1	Clinical strain derived from e03.04 isolate	<0.015	0.03–0.06	0.0005–0.001	64–128	0.0005–0.001	P1B3	5187
NG3.2	Clinical strain derived from e03.04 isolate	0.03–0.06	0.03–0.06	0.0005–0.001	64–128	0.001–0.002	P1B3	5187
NG7	Clinical strain, using as a recipient for spot-transformation	0.12–0.25	0.015	0.001	16–32	0.002	P1B3	8633
NG94	Clinical strain, using as a recipient for spot-transformation	0.015–0.03	<0.03	0.001	16–32	<0.002	P1B3	8401

*PEN, penicillin; TET, tetracycline; CIP, ciprofloxacin; SPT, spectinomycin; CRO, ceftriaxone*.

For any manipulations, gonococci were cultivated on the BBL™ GC agar base (Becton Dickinson & Co, USA) supplemented with 1% BBL™ IsoVitaleX™ Enrichment (Becton Dickinson & Co, USA) at 37°C in a humid atmosphere with 5% CO_2_ during 20–24 h.

### Phenotypic characterization

Susceptibility testing to penicillin G, tetracycline, ciprofloxacin, spectinomycin and ceftriaxone was performed by agar dilution method in accordance with CLSI recommendations (CLSI document M100-S17, 2007). All antibiotics were manufactured by Sigma, USA. *N. gonorrhoeae* strain ATCC 49226 was used as a control.

### Gonococcal transformation

Spot-transformation of gonococci was performed as described (Dillard, 2011). Briefly, 200 ng of amplified DNA fragments in a 10 μl volume were placed into spots (diameter approximately 1.5 cm) on a pre-warmed (37°C) GC base agar plate. The spot was allowed to soak into the plate. Then piliated colonies of *N. gonorrhoeae* recipient strains were streaked across the plate through the DNA spots. After overnight incubation at 37°C in a humid atmosphere with 5% CO_2_, colonies were swabbed from the spot with a Dacron swab and re-suspended in 100 μl of storage medium (50% BBL™ Brain Heart Infusion, Becton Dickinson & Co, USA, 30% Fetal Bovine Serum, Gibco, USA, 20% glycerin, Sigma, USA). This suspension of cells was then diluted and plated on both GC base agar alone, and GC base agar supplemented with 64 mg/L of spectinomycin. These plates were incubated at 37°C in a humid atmosphere with 5% CO_2_ for one to two days, and individual transformants were picked up from spectinomycin supplemented plate. Selected colonies were cultivated once again on spectinomycin supplemented plates; the obtained *N. gonorrhoeae* strains were stored at −80°C. Transformation frequency was calculated as the number of spectinomycin-resistant colony-forming units (SPT^r^ CFU/ml) per total CFU (CFU/ml).

### Genotypic characterization uses few methods: PCR and sequencing and MALDI-TOF MS

Total genomic DNA from tested *N. gonorrhoeae* strains was isolated by “DNA express” kit (Lytech Ltd, Russia). When necessary, the prepared DNA samples were stored at a temperature of −20°C.

Conventional PCR was done using the genomic DNA as a template and oligonucleotide primers described in Table 2. All primers were newly designed using the Oligo_6.31 software (Molecular Biology Insights Inc., USA) and sequences of *N. gonorrhoeae* strain FA1090 (GenBank accession number is NC_002946). Amplification was carried out in 10 μl of 66 mM Tris-HCl pH 9.0; 16.6 mM (NH_4_)_2_SO_4_; 2.5 mM MgCl_2_; 0.2 mM of each dNTP; 5 pmol of each primer (Table 2) and 1 unit of TaqPolymerase (Fermentas, Lithuania) under the following conditions: 94°C for 20 s, 60°C for 20 s, and 72°C for 15 s, in 35 cycles. A programmed thermocycler TETRAD DNA ENGINE (MJ Research Inc.) was used. The amplification products were analyzed by electrophoresis in 2% agarose gel.

**Table 2:** **List of primers selected for PCR and sequencing of *N. gonorrhoeae* genes encoding 16S rRNAs, and ribosomal proteins S4, S5, and S8**.

**Genetic characterization of selected *N. gonorrhoeae* strains**					
**Target**		**Primers for PCR, 5′-3′ sequence (amplicon length)**		**Primers for sequencing, 5′-3′ sequence**	
1st copy of 16S rRNA		aaccgatgaccccctgcttg; gaaaaatgcatggcaggccg (2194bp)		aaccgatgaccccctgcttg	
2nd copy of 16S rRNA		aaccgatgaccccctgcttg; accgtaaccgaatgcccctg (2409bp)		tgacgtgtgaagccctggtcataa	
3rd copy of 16S rRNA		aaccgatgaccccctgcttg; gcaaacccatgacatttacc (2435bp)		acgctaccaagcaatcaagttgcc	
4th copy of 16S rRNA		aaccgatgaccccctgcttg; acgggatcgggaacgaaaac (2432 bp)		tcatcggccgccgatattggcaac	
*RPS4 (rpsD* gene)		tattaaaggtccaggtccaggtcg; catggatgacagtaagatacggcg (954bp)		as for PCR	
*RPS5 (rpsE* gene)		cgtgcacatgcacgatgagattca; tgagaaggctaaagcagcaggtgt (727bp)		as for PCR	
*RPS8 (rpsH* gene)		tcaatccattcctcgtaatgcggc; tttacttctacaccagcgggaacc (637bp)		as for PCR	
Genomic DNA fragment for spot transformation		tcaaagcaactgtaggtcgg; tgcgttctggtgctaaaggc (12931bp)		
**Identification of RPS5 Thr-24→Pro mutant strains**					
Target	Primers for PCR, 5′-3′ sequence (amplicon length)	Primer for primer extension (minisequencing) reaction, 5′-3′ sequence, molecular weight	Composition of dNTP and ddNTP mix	Predicted molecular weight of primer extension reaction products for wild type	Predicted molecular weight of primer extension reaction products for mutant type
A70C mutation in *rpsE* gene	cgtgcacatgcacgatgagattca; tgagaaggctaaagcagcaggtgt (727 bp)	gacgacctttaactactttgg (6397 Da)	dG, ddT	6685 Da (primer + ddT)	7014 Da (primer + dG +ddT)

*The scheme of identification of A70C mutation in rpsE gene by primer extension reaction followed by mass spectrometry measuring is also presented*.

For further sequencing analysis dephosphorylation of the 5′-end phosphate groups of dNTPs and cleavage of primers in the post-amplification reaction mixture was done by incubation with 0.5 U of shrimp alkaline phosphatase and 0.1 U of exonuclease I (both enzymes from Fermentas, Lithuania) for 20 min at 37°C, followed with inactivation by heating at 85°C for 10 min. Sequence analysis of 16S rRNA genes as well as genes cording the ribosomal proteins S5, S4, and S8 was performed with dedicated primers (Table 2) by the modified Sanger method using the ABI Prism™ BigDye® Terminator v. 3.1 Cycle Sequencing Kit and 3730xl DNA Analyzer (Applied Biosystems, USA) according to the manufacturer's instruction. Analysis of the nucleotide sequences as well as deduced amino acid sequences was done using the Vector NTI Advance v. 9.0 software (Infomarks Incorporation, USA).

*N. gonorrhoeae* multi-antigen typing (NG–MAST) was performed as originally described (Martin et al., 2004). Sequence types were identified via NG-MAST (http://www.ng-mast.net) websites.

The rapid identification of A70C nucleotide substitution in *rpsE* gene, leading to Thr-24→Pro amino acid mutation in RPS5, was done by primer extension reaction followed by MALDI ToF mass spectrometry. For this, PCR was performed as mentioned above with primers form Table 2. The dephosphorylation of the 5′-end phosphate groups of dNTPs in the post-amplification reaction mixture was carried out by incubation with 0.5 U of shrimp alkaline phosphatase (Fermentas, Lithuania) for 20 min at 37°C, followed by inactivation of the enzyme by heating for 10 min at 85°C. After that amplicon was used as template in thermocyclic primer extension reaction, carried out in 20 μ L of the mixture of 66 mM Tris-HCl pH 9.0; 16.6 mM (NH_4_)_2_SO_4_; 2.5 mM MgCl_2_; 0.2 mM of each dGTP and ddTTP, 10 pmol of internal primer (see Table 2) and 2 units of TermiPol DNA Polymerase (Solis Biodyne, Estonia) according to the followed profiling: 94°C for 20 s, 58°C for 20 s, and 72°C for 15 s, in 70 cycles.

The products from primer extension reaction were purified using the SpectroCLEAN Kit (Sequenom, USA) according to the manufacturer's instruction.

An aliquote (0.2–1 μL) of purified products from primer extension reaction was spotted onto a matrix, which was preliminarily dried on the MALDI target AnchorChip™ with 400 μm diameter of anchor sports (Bruker Daltonics, Germany). The matrix employed was a saturated solution of 3-hydroxypicolinic acid (Fluka, Germany) in a 1:1 acetonitrile/water mixture (Merck, Germany) mixed with 0.4 M dibasic ammonium citrate (Fluka, Germany) in 9:1 vol. ratio. All solvents were of quality suitable for mass spectrometry. Mass spectra were collected on a Microflex LT MALDI ToF mass spectrometer (Bruker Daltonics, Germany) that was operated in the positive linear mode. A 337 nm nitrogen laser with the 9 Hz pulse frequency was used. Mass spectrometer parameters were optimized for the range of m/z values from 1000 to 10,000, using a peptide standards set for calibration. Each mass spectrum was collected at 30 laser pulses at constant laser power and constant threshold value, in order to enhance the resolution.

### Accession numbers

DNA sequences of *rspE* gene alleles of *N. gonorrhoeae* strains NG3, NG3.1, and NG3.2 were deposited into GenBank [accession numbers KF021591, KF021592, and KF021593, respectively].

## Results and discussion

In this study we identified a molecular mechanism responsible for spectinomycin resistance in gonococci. As a part of unrelated experiment, *N. gonorrhoeae* strains with Spt-R phenotype were occasionally isolated. They were derived from clinical isolate e03.04 belonged to Por1B3 serotype. Collected in 2004 it was identified as susceptible to penicillin, tetracycline, ciprofloxacin, and ceftriaxon. Spectinomycin MIC was defined as 32-64 mg/L, which corresponded to intermediate resistant (Spt-I) phenotype. When the susceptibility profile of this isolate was retested in 2008 two colonies emerged on the plate supplemented with 64 mg/L of spectinomycin. Each colony was seeded once again on plates with the same antibiotic concentration, and then on the plates with 128 mg/L of spectinomycin. It was a poor growth on plates with 128 mg/L of spectinomycin, so two clones grown well under the 64 mg/L of spectinomycin were stored.

It should be noted that neither one of other *N. gonorrhoeae* isolates tested in this experiment nor the same e03.04 clinical isolate examined in repeats revealed any additional colonies survived under 64 mg/L of spectinomycin. Thus we have considered two occasionally isolated Spt-R clones as a small fraction of heterogeneous e03.04 clinical isolate. The collected strains were designated as NG3.1 and NG3.2. Gonococci derived from e03.04 isolate susceptible to spectinomycin treatment were also collected and stored as NG3 *N. gonorrhoeae* clinical strain.

Susceptibility testing data for clinical e03.04 isolate as well as for selected *N. gonorrhoeae* strains is presented in Table 1. In accordance with NG-MAST scheme all of them belonged to the same 5187 sequence type that supported the assumption of a common origin of the strains studied. Since, the only previously reported mechanism for Spt-R gonococci was C1192T mutation in 16S rRNA gene (Maness et al., 1974; Galimand et al., 2000), we have sequenced all four copies of 16S rRNA genes located in *N. gonorrhoeae* genomic DNA (for the number of rRNA genes copies see rrnDB database, http://ribosome.mmg.msu.edu/rrndb/index.php). For each tested strain, all four copies of 16S rRNA genes were found identical. Indeed, the 1192 position as well as a locus encoding for the 34 helix of 16S rRNA, which forms the spectinomycin-binding site of ribosome (Carter et al., 2000; Wirmer and Westhof, 2006) possessed the wild-type sequence in NG3 strain as well as in NG3.1 and NG3.2 ones. So, we have proposed that other molecules located close by to the ribosomal spectinomycin-binding site could be involved in formation of Spt-R phenotype.

**Figure 1:**
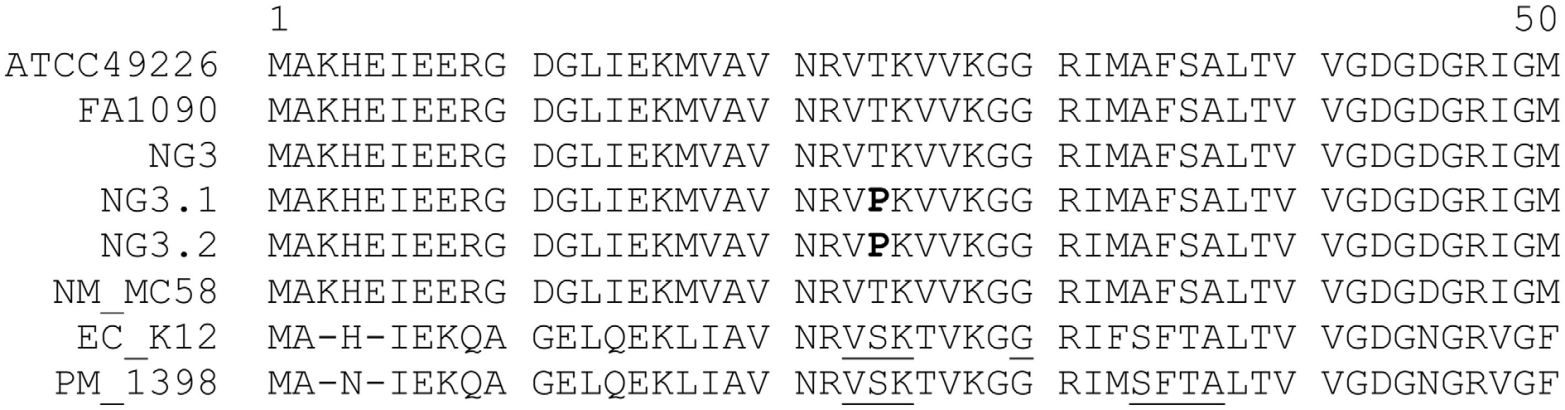
**Multiple alignment of RPS5 sequences of NG3, NG3.1, and NG3.2 strains**. The Thr-24→Pro mutation is bolded. Deduced amino acid sequences of RPS5 of wild-type *N. gonorrhoeae* (ATCC49226, FA1090), *N. meningitidis* (NM_MC58), *E. coli* (EC_K12), and *P. multocida* (PM_1398) are also presented. Regions involved in formation of resistance to spectinomycin are underlined [according to Davies et al. (1998); Kehrenberg and Schwarz (2007)].

The crystal structure of the 30S subunit of bacterial ribosome from *E. coli* in complex with spectinomycin (Carter et al., 2000) was taken into account and the ribosomal proteins S4, S5, and S8 were picked up for further examination due to their vicinity to a spectinomycin-binding site. Accordingly, *rpsD*, *rpsE*, and *rpsH* genes were amplified and sequenced. No differences were found between *rpsD*, and *rpsH* genes. However, both Spt-R *N. gonorrhoeae* strains (NG3.1 and NG3.2) carried the same A70C mutation in *rpsE* genes, leading to Thr-24→Pro amino acid substitution at RPS5, which mapped to Ser-22 residue of RPS5 from *E. coli* (Figure 1).

The 19–33 loop of RPS5 is known to be RNA binding site, and is the most frequent zone for location of mutations related to spectinomycin resistance (Funatsu et al., 1972; Wirmer and Westhof, 2006; Kehrenberg and Schwarz, 2007). Mostly, RPS5 mutations associated with Spt-R phenotype occur simultaneously with 16S rRNA changes. However, the isolated Ser-22→Pro RPS5 mutation in resistant to spectinomycine *E. coli* strain was also described (Wilcox et al., 2001). Basing on this knowledge, we have suggested that the Thr-24→Pro substitution found in RPS5 from NG3.1 and NG3.2 *N. gonorrhoeae* strains confer the Spt-R phenotype.

As an argument, that the single Thr-24→Pro mutation in RPS5 can support a Spt-R phenotype alone, we performed a transformation of spectinomycin sensitive *N. gonorrhoeae* strains by the exogenic DNA fragment contained mutant *rpsE* gene. Furthermore, the experiments described below demonstrated a potential role of HGT in spread of spectinomycin resistance.

The 12931 bp fragments of *N. gonorrhoeae* chromosomal DNA, which include whole *rpsE* gene and the nearest DNA uptake sequences (DUS12, 5′ atgccgtctgaa 3′) on their ends were amplified with primers indicated in Table 2. It was done using the NG3 (wild type) and NG3.1 (mutant) genomic DNAs as templates. The presence of DUS12 is known to be necessary for DNA recognition and uptake and therefore, mediates an increase in transformation efficiency (Duffin and Seifert, 2010).

**Figure 2:**
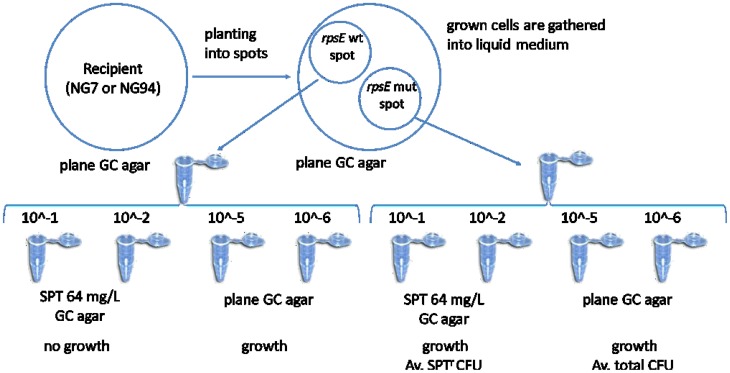
**The principal scheme of sport transformation**. Amplified DNA fragments carried mutant (mut) and wild type (wt) of *rpsE* gene cording the ribosomal protein S5 (RPS5) were placed in spots on a GC base agar plate. Then piliated colonies of recipient strains (NG7 and NG94) were streaked across the plate through the DNA spots. After overnight incubation, bacterial cells were swabbed from the spots into a liquid medium, diluted and plated on both GC base agar alone, and GC base agar supplemented with 64 mg/L of spectinomycin (SPT). The individual transformants emerged on SPT-supplemented plates were picked up for further examination. Colony-forming units (CFUs) were counted on each plate.

Two different *N. gonorrhoeae* strains—NG7, and NG94, susceptible to spectinomycin were chosen as recipients. For each recipient, the spot transformation by purified amplified DNA fragment contained mutant (or wild) variant of *rpsE* gene was carried out. The principal scheme of an experiment is represented in Figure 2. The transformation of recipients by a DNA fragment carried a wild variant of *rpsE* gene was used as a negative control. Each experiment was repeated in triplicate.

As a result, 74.7 ± 5.7 and 15.6 ± 2.5 colonies emerged on GC agar plates supplemented with 64 mg/L of spectinomycin and derived from NG7 and NG94 bacterial cells transformed by mutant *rpsE* gene, respectively (Table 3). Bacterial cells transformed by DNA fragment carried wild type of *rpsE* gene formed no colonies on spectinomycin supplemented GC agar plates. Transformation frequency calculated as the number of SPT^r^ CFU per total CFU was found 2.2 ± 0.4 × 10^−5^ and 1.7 ± 0.2 × 10^−5^ for NG7 and NG94 *N. gonorrhoeae* strains, respectively.

**Table 3:** **The calculated results of transformation experiments**.

**Recipient**	**Av. SPT^r^ CFUs**	**Av. SPT^r^ CFU/ml**	**Av total CFU/ml**	**Transformation efficiency**
NG7	74.7 ± 5.7	3.5 ± 0.4 × 10^4^	1.6 ± 0.5 × 10^9^	2.2 ± 0.4 × 10^−5^
NG94	15.6 ± 2.5	0.8 ± 0.2 × 10^4^	4.7 ± 0.2 × 10^8^	1.7 ± 0.2 × 10^−5^

*An average data from three replicas is presented*.

Each transformant survived after a re-cultivation on spectinomycin supplemented (64 mg/L) agar plates was tested by sequencing. All tested strains carried A70C mutation in *rpsE* gene identical to NG3.1 and NG3.2 *N. gonorrhoeae* strains and leading to Thr-24→Pro amino acid substitution in RPS5. Otherwise, in accordance with NG MAST, they belonged to the same sequence types as the recipient strains from which they originated.

The spectinomycin MICs were measured for the transformants derived both from NG7 and NG94 *N. gonorrhoeae* recipient strains. In all cases, spectinomycin MICs increased fourfold (from 16–32 mg/L to 64–128 mg/L) and became identical to the ones of NG3.1 and NG3.2 *N. gonorrhoeae* strains.

Described experiments verified that single Thr-24→Pro amino acid substitution in RPS5 confers the Spc-R phenotype of *N. gonorrhoeae* strains.

Furthermore, the transformation efficiency was found relatively high (ca. 10^−5^) and was not statistically different between both recipient strains. This finding supports the statement that the HGT through the natural transformation permits movement of mutant allele of *rpsE* gene and increases the opportunity for the spread of spectinonycin resistance within gonococcal population.

The RPS5 mutation we verified here appeared to be the same that was described for *N. gonorrhoeae* WHO A strain (Unemo et al., 2013). This mutation results in low-level spectinomycin resistance in gonococci that agrees with our conclusion.

The main issue is that in this study, conducted independently the mutant allele of *rpsE* gene was found in clinical *N. gonorrhoeae* strain isolated in Russia. This emphasizes that the described molecular mechanism of Spt-R phenotype formation is a universal one and spreads worldwide.

To extend the knowledge of frequency of RPS5 Thr-24→Pro mutants the entire laboratory collection of *N. gonorrhoeae* genomic DNA (*n* = 1131) acquired since 2002–2008 years was analyzed. The majority of them (*n* = 1113) were susceptible to spectinomycin, and the 18 strains revealed the Spt-I phenotype (MICs = 32–64 mg/L). Keeping in mind that NG3.1 and NG3.2 strains possessed Thr-24→Pro mutation in RPS5 were found occasionally as a small fraction of a wild population, we did not exclude susceptible to spectinomycin gonococci from the examination.

No more mutant *N. gonorrhoeae* strains have been found. It seems the tested Thr-24→Pro substitution in RPS5 is very rare among gonococci. This may reflect the uncommon use of spectinomycin in therapy of gonorrhea. On the other hand, we cannot deny that gonococci with mutation in ribosomal protein could be somehow defective in their survival and population spreading. Undoubtedly, fitness cost due to such mutation requires further careful investigation.

Nevertheless, our study is an additional evidence of the existing of clinical *N. gonorrhoeae* strains with moderate spectinomycin resistance due to isolated mutations in RPS5. It is very important in terms of the surveillance and the therapy of gonococcal infection. The extended knowledge of the potential mutations responsible for resistant phenotypes is essential in the development of new tests suitable in clinical setting, which aim to establish the correct antimicrobial therapy.

## Conflict of interest statement

The authors declare that the research was conducted in the absence of any commercial or financial relationships that could be construed as a potential conflict of interest.
